# Molecular Genetics and Interferon Signature in the Italian Aicardi Goutières Syndrome Cohort: Report of 12 New Cases and Literature Review

**DOI:** 10.3390/jcm8050750

**Published:** 2019-05-26

**Authors:** Jessica Garau, Vanessa Cavallera, Marialuisa Valente, Davide Tonduti, Daisy Sproviero, Susanna Zucca, Domenica Battaglia, Roberta Battini, Enrico Bertini, Silvia Cappanera, Luisa Chiapparini, Camilla Crasà, Giovanni Crichiutti, Elvio Dalla Giustina, Stefano D’Arrigo, Valentina De Giorgis, Micaela De Simone, Jessica Galli, Roberta La Piana, Tullio Messana, Isabella Moroni, Nardo Nardocci, Celeste Panteghini, Cecilia Parazzini, Anna Pichiecchio, Antonella Pini, Federica Ricci, Veronica Saletti, Elisabetta Salvatici, Filippo M. Santorelli, Stefano Sartori, Francesca Tinelli, Carla Uggetti, Edvige Veneselli, Giovanna Zorzi, Barbara Garavaglia, Elisa Fazzi, Simona Orcesi, Cristina Cereda

**Affiliations:** 1 Department of Brain and Behavioral Sciences, University of Pavia, 27100 Pavia, Italy; jessica.garau@mondino.it (J.G.); anna.pichiecchio@mondino.it (A.P.); simona.orcesi@mondino.it (S.O.); 2 Genomic and Post-Genomic Center, IRCCS Mondino Foundation, 27100 Pavia, Italy; valentemarialuisa80@gmail.com (M.V.); daisy.sproviero@mondino.it (D.S.); susanna.zucca@unipv.it (S.Z.); camillacrasa@tiscali.it (C.C.); 3 Unit of Child and Adolescence Neurology, IRCCS Mondino Foundation, 27100 Pavia, Italy; cavallerav@gmail.com (V.C.); valentina.degiorgis@mondino.it (V.D.G.); 4 Pediatric Neurology Unit, V. Buzzi Children’s Hospital, 20154 Milan, Italy; davide.tonduti@asst-fbf-sacco.it; 5 Child Neuropsichiatry, Department of Woman and Child Health and Public Health, Fondazione Policlinico Universitario A. Gemelli IRCCS, Università Cattolica del Sacro Cuore, 00168 Roma, Italy; domenicaimmacolata.battaglia@unicatt.it; 6 Department of Developmental Neuroscience, IRCCS Stella Maris Foundation, 56128 Pisa, Italy; r.battini@inpe.unipi.it (R.B.); francesca.tinelli@inpe.unipi.it (F.T.); 7 Unit of Neuromuscular and Neurodegenerative Disorders, Bambino Gesù Children’s Hospital, IRCCS, 00165 Rome, Italy; ebertini@gmail.com; 8 S.O.D. Neuropsichiatria Infantile, Ospedali Riuniti "G. Salesi", 60123 Ancona, Italy; silvia.cappanera@gmail.com; 9 Neuroradiology Unit, Fondazione IRCCS Istituto Neurologico Carlo Besta, 20133 Milan, Italy; luisa.chiapparini@istituto-besta.it; 10 Clinica Pediatrica ASUIUD, 33100 Udine, Italy; crichiutti.giovanni@aoud.sanita.fvg.it; 11 Child Neurology Unit, IRCCS, Santa Maria Nuova Hospital, 42123 Reggio Emilia, Italy; dellagiustina.elvio@asmn.re.it; 12 Developmental Neurology Division, Fondazione IRCCS Istituto Neurologico Carlo Besta, 20133 Milan, Italy; Stefano.Darrigo@istituto-besta.it (S.D.); veronica.saletti@istituto-besta.it (V.S.); 13 Child Neurology and Psychiatry Unit, ASST Spedali Civili of Brescia, 25123 Brescia, Italy; micaela.desimone@gmail.com (M.D.S.); elisa.fazzi@unibs.it (E.F.); jessica.galli@unibs.it (J.G.); 14 Department of Clinical and Experimental Sciences, University of Brescia, 25123 Brescia, Italy; 15 Department of Neuroradiology andLaboratory of Neurogenetics of Motion, Montreal Neurological Institute and Hospital, McGill University, Montreal, QC H3A2B4, Canada; roberta.lapiana@mail.mcgill.ca; 16 Child Neurology Unit, IRCCS Istituto delle Scienze Neurologiche, 40139 Bologna, Italy; tullio.messana@ausl.bologna.it (T.M.); antonella.pini@isnb.it (A.P.); 17 Department of Pediatric Neuroscience, Fondazione IRCCS Istituto Neurologico Carlo Besta, 20133 Milan, Italy; Isabella.Moroni@istituto-besta.it (I.M.); nardo.nardocci@istituto-besta.it (N.N.); Giovanna.Zorzi@istituto-besta.it (G.Z.); 18 Medical Genetics and Neurogenetics Unit, Movement Disorders Diagnostic Section, Fondazione Irccs IstitutoNeurologico Carlo Besta, 20133 Milan, Italy; celeste.panteghini@istituto-besta.it (C.P.); barbara.garavaglia@istituto-besta.it (B.G.); 19 Department of Pediatric Radiology and Neuroradiology, V. Buzzi Children’s Hospital, 20154 Milan, Italy; cecilia.parazzini@asst-fbf-sacco.it; 20 Neuroradiology Unit, IRCCS Mondino Foundation, 27100 Pavia, Italy; 21 Unit of Child Neurology and Psychiatry, University Hospital Città della Salute e della Scienza, 10126 Turin, Italy; federica.ricci@unito.it; 22 Clinical Department of Pediatrics San Paolo Hospital - ASST Santi Paolo Carlo, 20142 Milano, Italy; elisabetta.salvatici@ao-sanpaolo.it; 23 Molecular medicine, IRCCS Fondazione Stella Maris, 56128 Pisa, Italy; filippo.santorelli@fsm.unipi.it; 24 Paediatric Neurology and Neurophysiology Unit, Department of Women’s and Children’s Health, University Hospital of Padua, 35128 Padua, Italy; stefano.sartori@unipd.it; 25 Neuroradiology Unit, Department of Radiology, ASST Santi Paolo e Carlo, San Carlo Borromeo Hospital, 20153 Milan, Italy; carla.uggetti@gmail.com; 26 Child Neuropsychiatry Unit, IRCCS Giannina Gaslini Institute DINOGMI, University of Genoa, 16147 Genoa, Italy; edvigeveneselli@gaslini.org

**Keywords:** Aicardi-Goutières Syndrome, Next Generation Sequencing, Interferon signature

## Abstract

Aicardi-Goutières syndrome (AGS) is a genetically determined early onset encephalopathy characterized by cerebral calcification, leukodystrophy, and increased expression of interferon-stimulated genes (ISGs). Up to now, seven genes (*TREX1, RNASEH2B, RNASEH2C, RNASEH2A, ADAR1, SAMHD1, IFIH1*) have been associated with an AGS phenotype. Next Generation Sequencing (NGS) analysis was performed on 51 AGS patients and interferon signature (IS) was investigated in 18 AGS patients and 31 healthy controls. NGS identified mutations in 48 of 51 subjects, with three patients demonstrating a typical AGS phenotype but not carrying mutations in known AGS-related genes. Five mutations, in *RNASEH2B*, *SAMHD1* and *IFIH1* gene, were not previously reported. Eleven patients were positive and seven negatives for the upregulation of interferon signaling (IS > 2.216). This work presents, for the first time, the genetic data of an Italian cohort of AGS patients, with a higher percentage of mutations in *RNASEH2B* and a lower frequency of mutations in *TREX1* than those seen in international series. *RNASEH2B* mutated patients showed a prevalence of negative IS consistent with data reported in the literature. We also identified five novel pathogenic mutations that warrant further functional investigation. Exome/genome sequencing will be performed in future studies in patients without a mutation in AGS-related genes.

## 1. Introduction

Aicardi-Goutières syndrome (AGS) is an early-onset rare genetic disorder with autosomal recessive or dominant inheritance. AGS patients usually demonstrate a congenital or sub-acute neurological involvement during their first year of life variably manifesting as microcephaly, spasticity, dystonia, seizures, cortical blindness and psychomotor delay [1]. Radiological findings commonly comprise cerebral calcification, white matter abnormalities and cerebral atrophy [2,3]. Furthermore, patients can show a variety of extraneurological symptoms, including chilblains, congenital glaucoma, raised levels of autoantibodies, hypothyroidism, insulin-dependent diabetes mellitus, Systemic Lupus Erythematosus (SLE), thrombocytopenia, hemolytic anemia, polygammaglobulinaemia, neonatal cardiomyopathy, bowel inflammation, demyelinating peripheral neuropathy, micropenis, and transitory antidiuretic hormone deficiency [1,2,3,4]. Later onset of the disease has also been described and is commonly characterized by a neurologic regression [2,5,6]. The classic phenotype is quite stereotyped, resembles congenital viral infections (TORCH complex) and other conditions in which cerebral calcification are associated with early onset encephalopathy [7].

An important feature of AGS is the presence of lymphocytosis and raised levels of interferon-alpha (IFN-α) [8,9], CXCL10 and CCL2 [10] in cerebrospinal fluid (CSF). IFN-α is thought to play a central role in the development of autoimmune diseases: SLE patients for example frequently show an increased expression of interferon-stimulated genes (ISGs) in peripheral blood, defined with the term “interferon signature” (IS) [11,12]. Considering that AGS patients have increased IFN-α concentration both in CSF and peripheral blood, and that some of them may develop an early-onset form of SLE, in 2013 Rice and colleagues proposed the IS as a possible biomarker of AGS. They confirmed an increased expression of ISGs, *IFI27*, *IFI44*, *IFIT1*, *ISG15*, *RSAD2* and *SIGLEC1* in peripheral blood of AGS patients compared with healthy controls [13].

Mutations in seven genes (*TREX1*, *RNASEH2B*, *RNASEH2C*, *RNASEH2A*, *ADAR1*, *SAMHD1*, *IFIH1*) have been defined as pathogenic for AGS to date [1]. All these genes encode for proteins involved in nucleic acids metabolism and sensing. In detail, *TREX1* encodes for the major mammalian 3′->5′ DNA exonuclease [14]; the Rnase H2 complex acts on RNA:DNA hybrids and removes ribonucleotides embedded in DNA in order to prevent an abnormal immune system activation [15]; *ADAR1* converts selected adenosine residues into inosine (A-to-I RNA editing) in double-stranded RNA (dsRNA) [16]; *SAMHD1* converts deoxynucleoside triphosphates to the constituent deoxynucleoside and inorganic triphosphate [17] and *IFIH1* encodes for one of two cytoplasmic sensors of viral double stranded RNA [18]. It is hypothesized that a reduction or loss of function of the AGS1-6 proteins leads to a pathological accumulation of nucleic acids and activation of innate immunity with the abnormal release of IFN-α, whilst mutations in *IFIH1* confer a gain of function of the sensor with consequent increased production of type I IFN [19].

Thanks to these genetic discoveries many children worldwide have had a genetic diagnosis, even if few data are available regarding the distribution of mutations in populations selected by nationality and pedigree.

In this paper we describe, for the first time, the genetic landscape of a large Italian cohort of 51 AGS patients from 49 families: Twelve Italian subjects have never been described before, whilst 39 have already been included in previously reported cohorts. Moreover, we aimed to describe the ISGs expression in a subgroup of patients to define a possible association between the interferon scores and genetic mutations within the Italian AGS cohort.

## 2. Materials and Methods

### 2.1. Patients

The Italian cohort here presented is composed of 51 Italian AGS patients (19 females and 32 males) (Table 1), from 49 families, with ages from 1 to 28 years. All patients are descendants of parents and grandparents of Italian nationality and Italian patients with even one foreign parent were excluded. The patients were diagnosed in different centers, according to clinical suggestion guided by defined criteria [7,20,21]. Clinical data were obtained from medical records and through direct clinical contact while genetic data were acquired retrospectively except for 12 new diagnoses (Table 1). Clinical onset was defined, according to Livingston [5], based on the age of onset of the first symptoms and signs of disease in: Prenatal/neonatal onset, infantile onset (onset presenting in the first few months of life) and later onset (beyond the first year of life). The severity of the clinical picture was assessed by deriving a score formed by the administration of three scales for the evaluation of motor and communication abilities: The Gross Motor Function Classification System (GMFCS) [22], the Manual Ability Classification System (MACS) [23] and the Communication Function Classification System (CFCS) [24]. The summary clinical score ranges from 3 (when gross motor, manual, and communication abilities are fully preserved), to 15 (associated with very severe disability). A clinical score ≥ 12 corresponds to severe disability, a score between 6 and 12 to moderate clinical disability and a score ≤ 6 to a mild clinical disability (Table 1).

This study was approved by the local ethics committee (approval n. 3549/2009 of 30/9/2009 and 11/12/2009, and n.20170035275 of 23/10/2017) of the IRCCS Mondino Foundation, Pavia, Italy and written informed consent was obtained from every participant or authorized relatives.

Interferon signature analysis was performed in 18 out of 51 Italian AGS patients at IRCCS Mondino Foundation.

### 2.2. Genetic Tests and Data Analysis

Blood samples from eight AGS patients (4 females and 4 males) were collected at IRCCS Mondino Foundation in vacutainers containing EDTA. Genomic DNA extraction was performed using a semi-automated method Maxwell^®^ 16 System DNA Purification (Promega, Madison, WI, USA). DNA was quantified with NanoDrop ND1000 UV-Vis Spectrophotometer and Qubit^®^ fluorometer (Thermo Scientific, Waltham, MA, USA).

The genetic tests have been performed in different centers since 2006 initially using Sanger sequencing and then Next Generation Sequencing (NGS). For Sanger sequencing, we used a standard protocol to amplify AGS-related genes coding exons using primers located in adjacent intronic regions from genomic DNA by polymerase chain reaction (PCR). All amplicons were screened by direct sequencing using Big-Dye Terminator v3.1 sequencing kit (Applied Biosystems) and ABI 3130 Genetic Analyzer (Applied Biosystems, Foster City, CA, USA). Each fragment was sequenced on both strands. The alignment to the corresponding reference sequence was performed using Sequencher 4.8 software (Gene Codes Corporation, Ann Arbor, MI, USA). Now we present 12 new genetic diagnoses using Nextera Enrichment Sample Illumina (Illumina), according to the manufacturers’ instructions. Five diagnoses were performed at IRCCS Mondino Foundation, 5 at Besta Foundation and 2 at Bambino Gesù Children’s Hospital. In the panel were included *TREX1*, *RNASEH2B*, *RNASEH2C*, *RNASEH2A*, *ADAR1*, *SAMHD1*, *IFIH1* and *RNASET2* genes. DNA processing and DNA-seq analysis were carried out using Illumina MiSeq Sequencer. Samples were loaded on MiSeq instrument and the first steps of bioinformatic analysis (including base calling and demultiplexing) performed using MiSeq provided software (Real Time Analysis RTA v.1.18.54 and Casava v.1.8.2, Illumina, Inc., San Diego, CA, USA). FastQ files provided for each sample, containing mate paired-end reads after demultiplexing and adapter removal, were used as input for an ad-hoc developed pipeline previously described [36]. Variant annotation was performed using Annovar software (table_annovar.pl). Mutations were considered pathogenic if they were very rarely found in healthy controls (i.e., dbSNP, and 1000 Genomes databases), predicted to alter the sequence of the encoded protein (nonsynonymous, nonsense, splice-site, frameshift, and insertion/deletion mutations) and to adversely affect protein function, with the use of in silico prediction software (SIFT, PolyPhen, MutationTaster). Genetic regions with low coverage (less than 30×) and all identified variants were confirmed using Sanger sequencing (primer sequences and PCR conditions are available upon request).

### 2.3. Multiplex Ligation-Dependent Probe Amplification (MLPA)

In order to evaluate possible duplications or deletions, AGS-related genes were analyzed with SALSA MLPA P388-A2 Aicardi-Goutières syndrome probe mix kit (MRC-Holland, Amsterdam, The Netherlands). It consists of probes mapped on *TREX1*, *RNASEH2A*, *RNASEH2B*, *RNASEH2C* and *SAMHD1* genes. To perform data analysis, we used GeneScan ver.3.1. Data importation and elaboration have been made with the software Coffalyser (MRC-Holland, Amsterdam, the Netherlands). The changes were considered significant for values that presented a deviation greater than 30% compared to controls.

### 2.4. Interferon Signature

Peripheral blood from 18 patients and 31 healthy controls were collected into PaxGene™ tubes (PreAnalytiX, Hombrechtikon, Switzerland) for RNA isolation. Tubes were kept at room temperature for 2 h and frozen at −80 °C within 24 h. RNA extraction was performed according to the manufacturer’s protocol and its concentration assessed using a NanoDrop ND1000 UV-Vis Spectrophotometer (Thermo Scientific, Waltham, MA, USA). For each sample we retrotranscripted 800 ng of RNA using kit iScript™ Reverse Transcription Supermix for RT-qPCR (Bio-Rad, Hercules, CA, USA). The expression analysis of six interferon-stimulated genes was performed using the TaqMan Universal PCR Master Mix (Applied Biosystems, Paisley, UK), and cDNA derived from 40 ng total RNA. The relative abundance of target transcripts was measured using TaqMan probes for *IFI27* (Hs01086370_m1), *IFI44L* (Hs00199115_m1), *IFIT1* (Hs00356631_g1), *ISG15* (Hs00192713_m1), RSAD2 (Hs01057264_m1), and *SIGLEC1* (Hs00988063_m1), and normalized to the expression level of *HPRT1* (Hs03929096_g1) and *18S* (Hs999999001_s1) as described by Rice and collaborators [13,14,15,16,17,18,19,20,21,22,23,24,25,26,27,28,29,30,31,32,33,34,35,36,37]. To perform this assay, the LightCycler 480 (Roche) was used. AGS patient data were expressed relative to the average of 31 healthy controls. The median fold change of the six ISGs of the 31 normal controls plus two SDs (+2 SD), was used to create a score as reported in Reference [26]. In our case it was 2.216. For each patient, relative quantification (RQ) (2−ΔΔCt) [38], i.e., the normalized fold change relative to the mean of each ISGs of the 31 controls, was calculated. The mean interferon score was given by the mean of the six genes and if it was above the score, it was designated as positive.

## 3. Results

**Figure 1:**
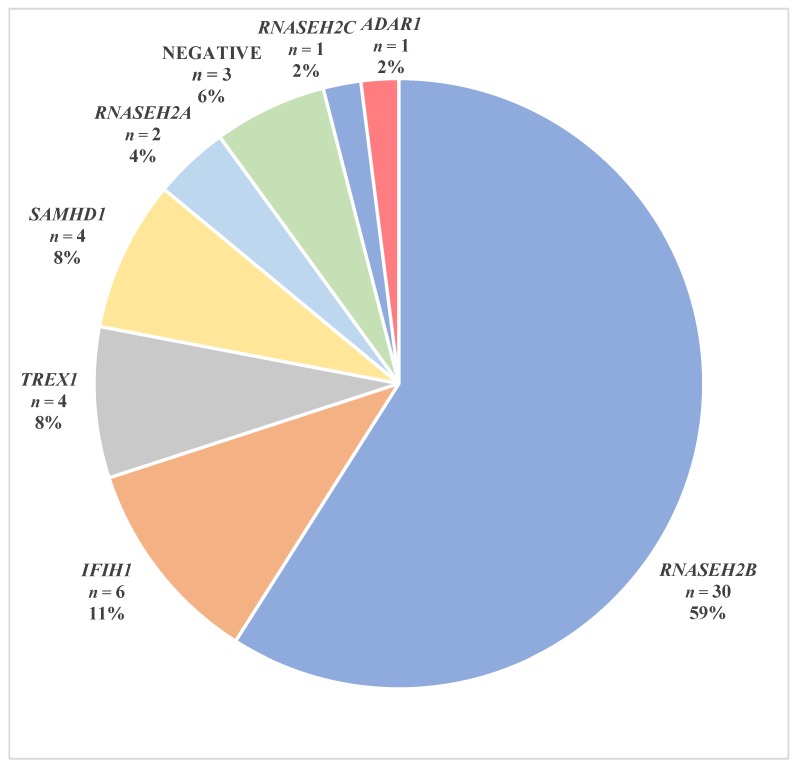
Numbers and percentages of patients with Aicardi–Goutières syndrome with or without mutations in *TREX1*, *RNASEH2A*, *RNASEH2B*, *RNASEH2C*, *SAMHD1*, *ADAR1* and *IFIH1* genes.

NGS genetic analysis identified mutations in one of the seven AGS-related genes in eleven of the twelve new patients, while thirty-nine patients were previously diagnosed of which three patients fulfilled the diagnostic criteria of AGS but did not carry mutations in AGS-related genes. Overall, mutations in one of the AGS-related genes were found in forty-eight out of fifty-one AGS patient (94%) as follows—59% of Italian AGS patients had mutations in *RNASEH2B*, 11% in *IFIH1*, 8% in *TREX1*, 8% in *SAMHD1*, 4% in *RNASEH2A*, 2% in *RNASEH2C* and 2% in *ADAR1* (Figure 1 and Table 2).

### 3.1. RNASEH2B Variants

Mutations in *RNASEH2B* were identified in thirty AGS patients. In particular, seventeen AGS patients (of which four are unreported) were homozygous for the c.529G>A/p. (Ala177Thr) (rs75184679) missense mutation in the exon 7 of *RNASEH2B* (P7-23). This is the most frequent mutation associated with AGS [1] and results in the substitution of alanine 177 with a threonine, a non-polar with a polar amino acid. In our cohort, the neurological picture was typical in ten of our seventeen patients, characterized by the onset of features in the first year of life of cerebral calcification, leukodystrophy, severe spasticity, dystonia, and developmental delay. Four patients also had epilepsy. Ten patients showed extraneurological involvement with either chilblains, or recurrent fevers. Three patients had a later onset (within the second year of life), six showed a mild phenotype, with an overall clinical score ranging from 3 to 6 and five had a normal intelligence quotient (IQ). Two of them showed exclusively a mild hemiparesis with greater involvement of the lower limb. At neuroimaging, three mild patients showed atypical brain CT scans with either no calcification in two cases (CT scan at 16 months, 7 months after onset in one patient; and at 15 months, 4 months after onset, and follow up at 4 years in the second), or diffuse microcalcifications in one. Additionally, the second patient with no calcifications [21] had deep and periventricular white matter changes at the first MRI that normalized at follow-up. Seven patients (P24-30) were compound heterozygous for the p.(Ala177Thr) mutation and the c.488C>T/p.[Thr163Ile] (rs79310911) variant within exon 6 in the other allele. The polar amino acid threonine at position 163 is replaced by the non-polar isoleucine. These were all classic severe phenotypes, variably associated with epilepsy (three cases), extraneurological features (six cases, including one celiac disease), and infantile onset except for two patients who presented in the first month of life. The p.(Ala177Thr) variant was also associated with the substitution of tryptophan into a leucine at position 73 (c.218G>T/p.(Trp73Leu), rs78071087) in two siblings (P31–32). The onset of disease was in infancy in both, presenting with non-neurological features (transient chilblains in the context of familiarity for chilblains with onset in late childhood and adolescence). The baby girl showed normal early psychomotor development until age 16 months when, following an intercurrent infection the disease manifested with a progressive loss of independent walking. The younger brother showed a mild clinical picture until the age of 13 months when he evolved in a classic phenotype [31]. One patient (P34) in our cohort presented the c.554T>G/p.(Val185Gly) (rs74555752) homozygous variant where valine 185 is replaced by the small amino acid glycine. He showed a classic phenotype with epilepsy. Two patients (one unreported) were compound heterozygous for p.(Ala177Thr) and deletions of exons from 9 to 11 (P33) and splicing mutation c.64+1G>A (P36) respectively. Finally, a novel mutation p.(Ala212Val) was seen in association with the p.(Ala177Thr) variant in one patient (P35) and main prediction software described this variant as probably damaging (PolyPhen), damaging (SIFT) and disease causing (MutationTaster). All three patient had a classic severe phenotype with infantile onset.

### 3.2. TREX1 Variants

Four patients carried mutations in *TREX1*. The first patient (P1) presented a homozygous G to A transition (c.341G>A) within exon 1 of the *TREX1* gene (rs72556554). This transition leads to the amino acidic substitution from arginine 114 to a histidine, both amino acids being positively charged (p.(Arg114His)) the most common *TREX1* mutation worldwide. A second unreported patient (P4) was compound heterozygous for p.(Pro290_Ala295del) + p.(Arg114His) variants: The former is a deletion of 18 nucleotides whereas the latter results in the substitution of an arginine with a histidine. A third patient (P2) was compound heterozygous for c.262 ins AG/p.(Ser88Lysfs*) + c.290G>A/p.(Arg97His) (rs200773268) variants. The c.262 ins AG, p.(Ser88Lysfs*) variant results in the insertion of two nucleotides, which results in the addition of a premature stop codon within the transcript sequence. Consequently, this shortened form of *TREX1* protein is likely to be associated with a loss of function of the enzyme. In addition, c.290G>A results in an amino acid change from an arginine to a histidine [1]. The last patient (P3) presented a homozygous frameshift mutation c.150_151del/ p.(Gln51Glyfs*50) already been described [1]. All four patients experienced a neonatal onset of disease and a severe clinical course (accompanied by epilepsy in two cases). The second and third patients showed cardiomyopathy among other more typical extraneurological involvement (chilblains and glaucoma). The second patient, already described in Olivieri et al., had a notable immunological involvement which included thyroiditis, cANCA positivity, antiphospholipid antibodies and cerebral ischemia [25]: In addition to the “classic” leukoencephalopathy picture, brain MRI showed at 5 years of age a first ischemic event at the level of the left caudate nucleus and the follow up showed additional lacunar infarct outcomes and slight enlargement of the other homolateral malacic area. The last patient, never described in the literature, presented with pulmonary hypertension and sepsis, transient thrombocytopenia, atopic dermatitis and bilateral sensorineural hearing loss at birth.

### 3.3. RNASEH2A Variants

Two patients in our cohort were positive for mutations in the *RNASEH2A* gene., The first patient (P5) carried the compound heterozygous variants c.322C>T/p.(Arg108Trp) (rs76436818) + c.690C>A/p.(Phe230Leu) (rs79767407) placed in exons 3 and 7, respectively. The former variation results in the substitution of an arginine 108 with tryptophan, the latter is represented by the replacement of phenylalanine 230 with leucine. This patient showed a classic severe phenotype and experienced coeliac disease. The second (P6) patient harbored the p.(Arg186Trp) + p.(Val23Val). The first one is a missense mutation where a positively charged amino acid, arginine, is substituted with a non-polar one, tryptophan, the second one is a synonymous variation which is reported as pathogenic from Clinvar database since it creates a cryptic splice site resulting in an out of frame deletion. This patient, with a severe neonatal phenotype, died at 3 years of age and was already described by Rice and colleagues [26].

### 3.4. RNASEH2C Variants

Only one patient (P37) in our cohort was compound heterozygous for two mutations in *RNASEH2C*. We detected G to T transition in exon 1 leading to a substitution at position 39 of aspartic acid to a tyrosine (p.(Asp39Tyr)), a negatively charged and an aromatic amino acid respectively. This heterozygous mutation was seen in combination with a c.173-1G>C splice site variant [1]. The patient demonstrated a classic AGS phenotype with infantile onset, a severe spastic-dystonic tetraparesis and chilblains.

### 3.5. SAMHD1 Variants

*SAMHD1* mutations were found in four patients with three of these never described in the literature. Two siblings (P38-39) carried the p.(Asp137Gly) mutation. This family presents a mutation in exon 4, which results in the replacement of aspartic acid with glycine at position 137, two amino acids with different charge and dimensions. Main prediction software described this variant as probably damaging (PolyPhen), tolerated (SIFT) and disease causing (MutationTaster). Both parents are healthy carriers of this substitution whereas the two siblings are homozygous. They present a different clinical expression, albeit both with infantile onset: An 11 year old boy showed a classic severe phenotype with severe mental retardation, whilst a 9 year old girl exhibited a spastic paraparesis associated with mild cognitive impairment and no extraneurological involvement. Brain MRI showed a cystic leukoencephalopathy in the boy, while the girl showed multifocal leucoencephalopathy. Angio MRI was not performed in these patients, so we do not know if there is occult intracerebral large vessel involvement. One patient (P40) carries a c.1393C>T variation resulting in an early stop codon (p.(Gln465*)) in association with the splicing variant c.1410+5G>C. These variants, as well as the other one, has never been described in the literature and main prediction software described it as probably damaging (PolyPhen), damaging (SIFT) and disease causing (MutationTaster). The patient showed a classic AGS phenotype. The last child (P41) carries a homozygous deletion of exons 12−16, as previously described [1]. The boy presented with a severe clinical picture at 1 month, and imaging revealed brain calcifications and white matter hypodensities. At the age of 13 years the patient underwent MRI for the subacute onset of coma. Imaging showed temporoparietal intracerebral hematoma and angiography reveled three intracranial aneurysms [28].

### 3.6. ADAR1 Variants

We identified only a single Italian patient (P42) with disease, due to mutations in *ADAR1*. In this patient, we found the c.577C>G/p.(Pro193Ala) (rs145588689) the substitution was seen in association with the c.2608 G>A/p.(Ala870Thr) (rs398122893) variant in exon 8. The boy demonstrated a classic phenotype, presenting during the first months of life with increasing irritability, as well as sleep and feeding difficulties, associated with striatal necrosis [1,29].

### 3.7. IFIH1 variants

Six patients carried de novo heterozygous mutations in the *IFIH1* gene. Variant c.1178A>T/p.(Asp393Val) was observed within exon 6 in only one patient (P43) and leads to the replacement of a negatively charged amino acid, the aspartic acid, with a valine (non-polar) at position 393 [1,2,3,4,5,6,7,8,9,10,11,12,13,14,15,16,17,18,19,20,21,22,23,24,25,26,27,28,29,30]. Moreover, we identified in one patient (P44) the c.2471G>A/p.(Arg824Lys), a heterozygous transition which results in the substitution of arginine 824, a positively charged amino acid, with a lysine, positively charged [31]. In our group of patients the p.(Arg779His) was identified in two different patients (P45-46) (one of them, with classic severe infantile phenotype, never described) and p.(Arg720Gln) mutation in one patient (P47). These two variants cause the substitution of an arginine with histidine and glutamine, respectively. All three previous children presented with a classic severe phenotype with predominant infantile onset, but one of them, described in Galli et al. [31], presented normal head circumference, and normal non-verbal intelligence quotient. The last patient (P48), never described, carried the missense mutation c.2561T>A/p. [Met854Lys] in *IFIH1* which is described by main prediction software described as probably damaging (PolyPhen), damaging (SIFT) and disease causing (MutationTaster). Apart from the dermatologic features (erythematous and lentiginous cheeks, and hyperkeratotic lesions on elbows and knees), glaucoma and abnormal dentition with the delayed eruption of teeth, he showed a later onset of neurological disease at age 2 years. He exhibited a relatively mild neurological phenotype characterized by spastic paraparesis with a demyelinating sensory-motor polyneuropathy and preserved cognitive and language abilities. Neuroradiologic findings were normal until the age of 9 years, when a slight signal alteration of periventricular and deep frontal and parietal white matter was observed in brain MRI and CT showed cerebral calcification in bilateral globus pallidus, left putamen, bilateral frontal periventricular and deep subcortical white matter.

### 3.8. AGS Patients without Mutation in AGS-Related Genes

Three patients were phenotypically diagnosed with the infantile onset of AGS but no mutation in any of the AGS-related genes was identified. The first (P50) presented a severe spastic tetraparesis, hypopigmented lesions, and thyroiditis; the second (P51) with spastic diplegia, hypochromic lesions and transient chilblains. The third patient (P49) showed a severe classic AGS phenotype and clinical, biochemical and radiological findings all suggested a diagnosis toward this syndrome. After NGS, compound heterozygous mutations have been discovered in *RNASET2* (p.(Lys133del) + p.(Glu49*)), a gene involved in RNA metabolism in lysosomes [39] Both variants were predicted to be damaging by in silico analyses (Mutation Taster) [32].

### 3.9. Interferon Signature

**Figure 2:**
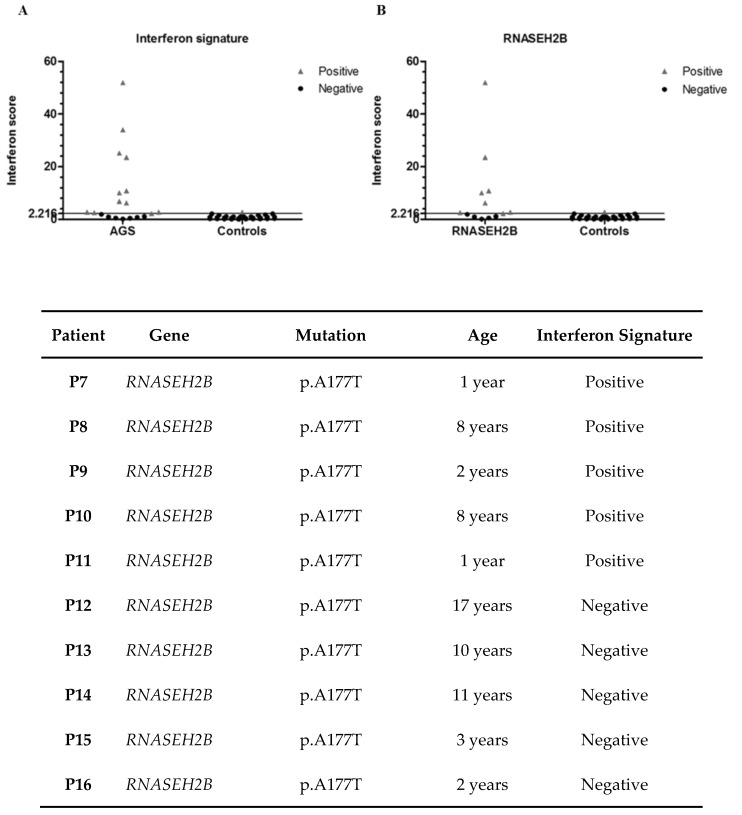

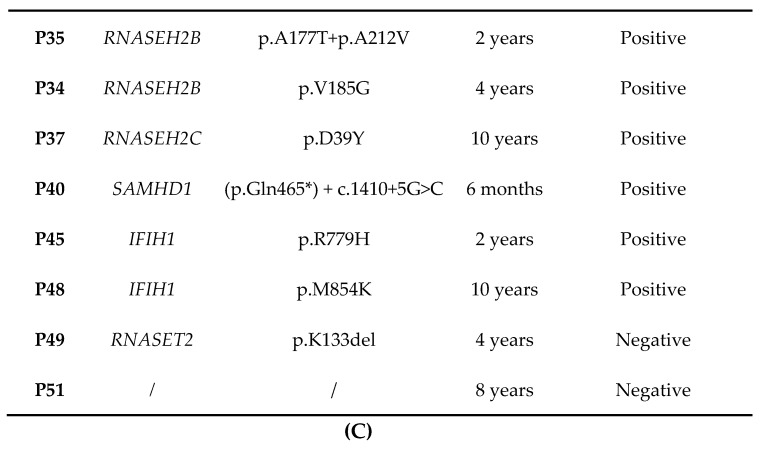
(**A**) Quantitative reverse transcription PCR of six ISGs *IFI27*, *IFI44*, *IFIT1*, *ISG15*, *RSAD2* and *SIGLEC1* in whole blood measured in 18 patients with Aicardi-Goutières syndrome and 31 controls. The threshold is calculated at 2.216: Higher values are considered positive, whereas lower scores are negative. (**B**) Interferon scores of 12 *RNASEH2B* mutated patients and 31 healthy controls. Scores above the threshold are positive whereas those below are negative. (**C**) Interferon signatures of 18 AGS patients.

**Figure 3:**
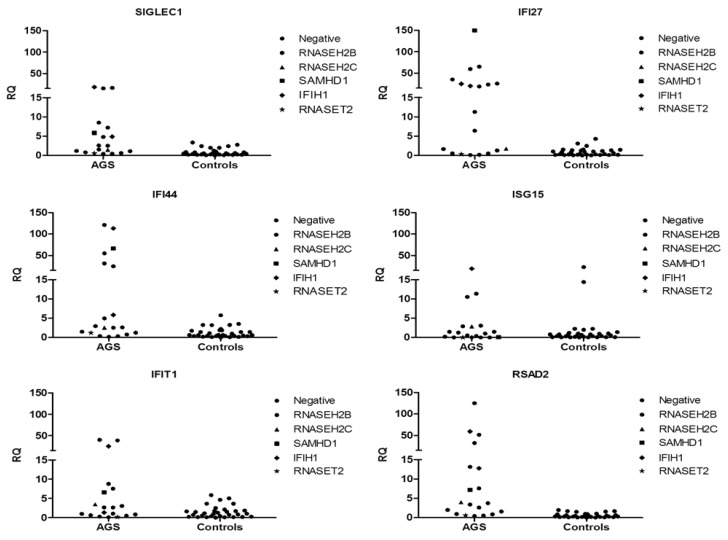
Median fold expression of six interferon-stimulated genes ISGs *IFI27*, *IFI44*, *IFIT1*, *ISG15*, *RSAD* and *SIGLEC1* according to the genotype of 18 AGS patients and controls.

We analyzed the expression levels of six ISGs *IFI27*, *IFI44L*, *IFIT1*, *ISG15*, *RSAD2* and *SIGLEC1* in a sub-group of eighteen AGS patients and thirty-one healthy controls (Figure 2C). The interferon signature of eleven out of eighteen patients was positive, whereas for six subjects was negative (Figure 2A and Figure 3). Among the eleven patients positive for an IS, seven were mutated in *RNASEH2B* (five with p.(Ala177Thr) variant, one presenting p. (Val185Gly) variation and one with p.(Ala177Thr) + p.(Ala212Val)), one in *RNASEH2C* (p.(Asp39Tyr) + c.173-1G>C substitutions), one in *SAMHD1* (p.(Gln465*) + c.1410+5G>C) and two in *IFIH1* (p.(Met854Lys) mutation and p.(Arg779His) variant). Regarding negative patients, five were mutated in *RNASEH2B* (all carrying p.(Ala177Thr) variation) (Figure 2B), one in *RNASET2* (p.(Lys133del) + p.(Glu49*)) and one presented all symptoms associated with AGS though the patient carried no mutations in the 7 AGS-related genes. Since this test is used for patients’ follow-up, we have been able to follow variations in ISG expression levels over time in seven patients. All patients had positive interferon scores at first measurement; later, only one *RNASEH2B* mutated patient showed a negative result, whereas the remaining six subjects had persistent positive signatures (data not shown).

## 4. Discussion

Aicardi-Goutières syndrome is an inherited subacute encephalopathy caused by mutations in *TREX1*, *RNASEH2B*, *RNASEH2C*, *RNASEH2A*, *SAMHD1*, *ADAR1*, *IFIH1*. All these AGS-related genes are involved in the nucleic acid metabolism or sensing, promoting, in physiological conditions, both the removal or identification of the presence of foreign nucleic acids. Mutations in *AGS 1-6* genes are hypothesized to lead to a loss of nucleic acids elimination and, consequently, their accumulation leads to the activation of an abnormal innate immune response, usually triggered by viral nucleic acids. Different situation with the same result is the case of *IFIH1*/*AGS7*, that encodes a protein (MDA5) that acts as a cytoplasmic "sensor" of the nucleic acids: If the gene is mutated, the sensor binds more “avidly” to the cytoplasmic RNA and causes excessive activation of the interferon response.

Up to now, AGS incidence worldwide is not well known and no correlation between variations and ethnicity has been proposed. Moreover, only a few papers describing the national cohort of AGS patients exist [40,41].

Here we report genetic data from an exclusively Italian cohort of fifty-one Italian AGS patients and our experience in assessing the IS in this disease.

Interestingly, the Italian AGS population presented different mutation percentages compared to the frequency in a previously reported larger, international cohort [1]: The most frequent mutations found in our cohort are in *RNASEH2B* and *IFIH1* genes (59% versus 36% globally, and 12% versus 3%, respectively), whereas the frequency of *TREX1* mutations was lower (8% versus 22%). The percentage of mutations in the remaining AGS-related genes is quite similar to one of the global AGS population.

The most frequent *RNASEH2B* mutation in our cohort is p.(Ala177Thr), as well as in the global AGS population [1]. In particular, this variant is well represented not only in homozygosity, but also in heterozygosity.

In the literature, the p.(Ala177Thr) mutation in a homozygous status is associated with an in vitro decrease in Rnase H2 stability, since it is able to modify the interactions between RNase H2 subunit b and subunit c [42]. This mutation could determine diminished RNase H2 levels and impaired cellular activity, both in vitro and in vivo correlation [43,44].

Regarding clinical features, variable phenotypes have been described in patients carrying the p.(Ala177Thr) mutation, both in homo and heterozygosity [45]. Moreover, mutations in *RNASEH2B* were in our cohort the ones mostly associated with heterogeneity in the clinical phenotype. This was even true in the ones carrying the p.(Ala177Thr) homozygous mutation: Ten patients (60%) showed a classic phenotype with or without extraneurological involvement, while six patients (35%) showed a mild phenotype that included normal IQ in all except one and mild hemiparesis in two. The onset of disease was predominantly within the first year of life, but later presentation was reported in three (18%).

Furthermore, compound heterozygous *RNASEH2B* mutated patients, carry the p.(Ala177Thr) mutation associated with the other three mutations. The p.(Ala177Thr) mutation combined with p.(Thr163Ile) variant leads to reduced stability and cellular levels of RNase H2, which results in an accumulation of ribonucleotidemonophosphates (rMNPs) embedded in genomic DNA. This represents an obstacle for replication fork progression and leads to genome instability and fork stalling [46]. Our patients with this genotype were all classic severe phenotypes, two (28%) showing neonatal onset. The same mutation associated with p.[Trp73Leu] variation is located at the hydrophobic core of B/C dimer and may lead to a decreased stability of the complex [42]. In our patients, this mutation was associated with the infantile onset of the extraneurological features and late onset neurological involvement [27].

At last, p.(Ala177Thr) has been observed in association with the novel mutation p.(Ex9-Ex11del). Exons 9-11 represent the C-terminus of the protein where the PCNA interacting protein-box sequence (PIP-box) is located. Interaction with PCNA, which is required to participate in DNA replication and repair, seems to be essential for RNase H2 function [47] and mutations in this site may impair their interplay.

Furthermore, our *RNASEH2B* mutated cohort of patients presents other homozygous variants. In particular, the p.(Val185Gly) mutation has already been described in the literature, first by Chon and colleagues who demonstrated that this genome alteration does not affect enzymatic complex catalytic activity, and it maintains the same action of wild-type protein [47]. Conversely, in 2011, this mutation was associated with possible impaired interactions with nucleic acid substrates or other proteins [43]. Our patient showed a classic phenotype with epilepsy.

Furthermore, our analysis allowed us to identify five novel mutations in *RNASEH2B*, *SAMHD1* and *IFIH1* genes. Regarding the novel mutation p.(Asp137Gly) in *SAMHD1* gene, main prediction software described this variant as probably damaging (PolyPhen) and disease causing (MutationTaster). Pathogenicity has also been correlated with p.(Ala177Thr) + p.(Ala212Val) and p.(Ala177Thr) + p.Ex9_Ex11del variations in the *RNASEH2B* gene, where once again the most frequent *RNASEH2B* variant, p.(Ala177Thr), is associated in heterozygosity with other mutations, p.[Ala212Val] and p.Ex9_Ex11del, respectively. Novel mutations have also been found in *SAMHD1* and *IFIH1* genes and both p.(Gln465*) + c.1410+5G>C (*SAMHD1*) and p.(Met854Lys) (*IFIH1*) mutations have been predicted to be damaging by the above-mentioned main prediction software.

Remarkably, we also present three patients with a complete AGS phenotype, but without mutations in the AGS-related genes. These cases well represent how general knowledge about AGS is still limited and how other genetic factors are likely to be involved in the pathogenesis of this disease. Whole exome sequencing analysis may reveal an effective tool to help the researcher in the discovering of other genes involved in AGS pathogenesis. Nonetheless, one patient presented two heterozygous mutations in *RNASET2*, a gene involved in ribosomal RNA metabolism and in the regulation of the immune response, both aspects usually altered in AGS patients [39,40,41,42,43,44,45,46,47,48]. The association between mutations in *RNASET2* gene and Aicardi-Goutières syndrome is still controversial, but few cases mutated in this gene have already been described in Reference [32].

Moreover, our analysis focused also on the action of IFN-α, since it plays an important and central role not only in autoimmune diseases, such as Systemic Lupus Erythematosus (SLE), but also in AGS. In as much as some AGS patients developed an early-onset form of SLE, Rice and colleagues adapted the so-called interferon signature from SLE to AGS patients [13].

We assessed the IS in eighteen AGS patients and thirty-one healthy controls. As reported in the IS data interferon signature chapter, eleven out of eighteen patients were positive, whereas the remaining seven subjects were negative. Among positive patients seven were mutated in *RNASEH2B* (38.9% out of the total percentage), one in *RNASEH2C* (5.5%), one in *SAMHD1* (5.5%), two in *IFIH1* (11%). The other seven negative patients were mutated in *RNASEH2B* (33.3%), *RNASET2* (5.5%) and one presents no mutations in the canonical AGS-related genes (5.5%). It is important to highlight that *RNASET2* mutated patient initially showed a positive IS [39] which progressively became negative.

If we exclusively take *RNASEH2B* mutated patients into consideration (thirteen on the whole) we can observe that eight were positive to the IS (61.5%) and five resulted negative (38.5%) (Figure 2B) in accordance with published data [1]. In fact, as previously reported [1], 31% of patients carrying mutations in *RNASEH2B* were negative to the IS, whereas 98% of patients mutated in other AGS-related genes was positive to this test. In particular, one patient has very higher levels of ISGs than all the other *RNASEH2B* mutated patients. Moreover, focusing on the most frequent *RNASEH2B* mutation (p.(Ala177Thr)), we saw a lack of correlation between mutation and interferon signature, since five out of thirteen patients had negative scores and the other eight were positive. Once again, there is a great variability not only among patients with mutations in different genes, but also among patients carrying variations in the same gene. At last, since subjects with mutations in the *RNASEH2B* gene are more likely to be negative for an IS, it may be interesting to study the molecular basis of this phenomenon. In addition, repeated measurements of IS at different time points, allowed us to observe variations of interferon scores in six patients. Two of them, who carry mutations in the *RNASEH2B* gene, initially had a positive score which later became negative.

The heterogeneity of our results casts doubts about considering the expression levels of ISGs as a biomarker of AGS, even if this test is certainly useful for diagnosis and to address genetic tests. Additional data are needed to better understand the role of ISGs on the pathogenesis and course of the disease. Hence, genetic analysis of AGS-related genes continues to be essential for the diagnosis of this syndrome.

## 5. Conclusions

In summary, this work considers for the first time the overall Italian cohort of AGS patients and opens the way to define an integrated manner to manage the patients considering the possible correlations between clinical, genetic, radiological and immunological factors. Several points remain unsolved, such as undefined patients, and the variability and role of ISG phenotypes. New approaches combining exome/genome sequencing and deep transcriptome analysis may help to diagnose AGS in difficult cases, such as patients with no mutations in AGS-related genes but with a clear AGS phenotype and mutated patients with a very mild phenotype.

## Figures and Tables

**Table 1:** Mutations and clinical features of 51 Aicardi-Goutières syndrome (AGS) patients.

Patient	Gene	Variant	Amino Acidic Substitution	Number of Patients (Gender)	Annotation *	Onset **	Clinical Score #	Clinical Phenotype	Epilepsy	GQ/IQ °	Chilblains and/or Recurrent Fevers	Other
P1	***TREX1***	c.341G>A	p.R114H	1 (Male)	Described [1]	Prenatal/neonatal	≥12	Spastic tetraparesis	Yes	Not evaluable	No	s-
P2	***TREX1***	c.262 ins AG + c.290G>A	p.S88Kfs* + p.R97H	1(Male)	Described [25]	Prenatal/neonatal	≥12	Spastic-dystonic tetraparesis	No	Not evaluable	No	Antiphospholipid syndrome, thyroiditis, cerebral ischemia
P3	***TREX1***	c.150_151del	p.N51Gfs*50	1 (Male)	Described [1]	Prenatal/neonatal	≥12	Spastic tetraparesis	No	Not evaluable	Yes	Cardiomyopathy
P4	***TREX1***	c.868_885del + c.341G>A	p.P290_A295del + p.R114H	1(Male)	Described [1]	Prenatal/neonatal	≥12	Spastic-dystonic tetraparesis	Yes	Not evaluable	Yes	Cardiomyopathy, pulmonary hypertension, sensoryneural hearing loss
P5	***RNASEH2A***	c.322C>T + c.690C>A	p.R108W + p.F231L	1(Female)	Described [1]	Prenatal/neonatal	≥12	Spastic-dystonic tetraparesis	No	Not evaluable	No	Celiac disease
P6	***RNASEH2A***	c.556C>T + c.69G>A	p.R186W + p.V23V	1 (Male)	Described [26]	Prenatal/neonatal	≥12	Spastic tetraparesis	Yes	Not evaluable	No	-
P7-P23	***RNASEH2B***	c.529G>A	p.A177T	17 (9 Males, 8 Females)	Described [1,21]	Infantile to later onset	4 patients ≤ 6 2 patients 6–12 11 patients≥ 12	Variable (Hemiparesis/Spastic diplegia/hypotonic-dystonic syndrome/Spastic-dystonic tetraparesis)	Yes (4 patients) No (13 patients)	Variable (from notevaluable to 90)	Yes/No	Variable neuroradiological features (brain calcification/no calcification/diffuse microcalcification/MRI normalization at follow up)
P24-P30	***RNASEH2B***	c.529G>A + c.488C>T	p.A177T + p.T163I	7 (2 Males, 5 Females)	Described [1]	Prenatal to infantile	≥12	Spastic-dystonic tetraparesis	Yes (3 patients) No (4 patients)	Variable (from not evaluable to >50)	No	Variable (no other features to celiac disease)
P31-P32^†^	***RNASEH2B***	c.529G>A + c.218G>T	p.A177T + p.W73L	2 (1 Male, 1 Female)	Described [27]	Infantile	6–12 (1 patient 11 1 patient 8)	Spastic-dystonic tetraparesis	No	1 patient <501 patient not evaluable	Yes	Variable neuroradiological features (brain calcification/ no calcification)
P33	***RNASEH2B***	c.529G>A +c.Ex9_Ex11del	p.A177T + p.Ex9_Ex11del	1 (Male)	Novel	Prenatal/neonatal	≥12	Spastic-dystonic tetraparesis	Yes	Not evaluable	Yes	-
P34	***RNASEH2B***	c.554T>G	p.V185G	1 (Male)	Described [1]	Prenatal/neonatal	≥12	Spastic-dystonic tetraparesis	Yes	Not evaluable	Yes	-
P35	***RNASEH2B***	c.529G>A + c.635C>T	p.A177T + p.A212V	1 (Female)	Novel	Infantile	≥12	Spastic-dystonic tetraparesis	No	Not evaluable	Yes	-
P36	***RNASEH2B***	c.529G>A + c.64+1G>A	p.A177T	1 (Male)	Described [1]	Infantile	≥12	Spastic tetraparesis	Yes	Not evaluable	Yes	-
P37	***RNASEH2C***	c.115G>T + c.173-1G>C	p.D39Y	1 (Male)	Described [1]	Infantile	≥12	Spastic-dystonic tetraparesis	No	Not evaluable	Yes	-
P38-P39^†^	***SAMHD1***	c.410A>G	p.D137G	2 (1 Male, 1 Female)	Novel	Infantile	1patient 6 1 patient ≥12	Spastic paraparesis/spastic-dystonic tetraparesis	No	1 patient 60 1 patient <50	Yes	-
P40	***SAMHD1***	c.1393C>T +c.1410+5G>C	p.Q465*	1 (Female)	Novel	Infantile	≥12	Spastic-dystonic tetraparesis	No	<50	Yes	-
P41	***SAMHD1***	Ex12_Ex16del	p.Ex12_Ex16del	1 (Male)	Described [28]	Infantile	≥12	Spastic-dystonic tetraparesis	Yes	<50	No	Cerebral vasculitis, three intracranial aneurysms
P42	***ADAR1***	c.577C>G + c.2608G>A	p.P193A + p.A870T	1 (Male)	Described [1,29]	Infantile	≥12	Spastic-dystonic tetraparesis	No	Not evaluable	No	Striatal necrosis
P43	***IFIH1***	c.1178A>T	p.D393V	1 (Male)	Described [1,30]	Later onset	≥12	Spastic-dystonic tetraparesis	Yes	Not evaluable	No	-
P44	***IFIH1***	c.2471G>A	p.R824K	1 (Male)	Described [31]	Infantile	≥12	Spastic-dystonic tetraparesis	No	Not evaluable	No	-
P45-46	***IFIH1***	c.2336G>A	p.R779H	2 (2 Males)	Described [1]	Infantile	≥12	Spastic-dystonic tetraparesis	No	Not evaluable	Yes	-
P47	***IFIH1***	c.2159G>A	p.R720Q	1 (Male)	Described [1]	Infantile	≥12	Spastic tetraparesis	Yes	Not evaluable	Yes	
P48	***IFIH1***	c.2561T>A	p.M854K	1 (Male)	Novel	Later onset	<6	Spastic paraparesis	Yes	97	No	Erythematous cheeks, lentiges, hyperkeratotic lesions, glaucoma, abnormal dentition, demyelinating sensory-motor polyneuropathy
P49	***RNASET2***	c.397_399delAAG + c.145G > T	p.K133del +p.E49*	1 (Male)	Described [32]	Infantile	≥12	Spastic-dystonic tetraparesis	No	Not evaluable	No	Cerebellar atrophy
P50	*/*	/	/	1 (Male)	/	Infantile	≥12	Spastic-dystonic tetraparesis	No	< 50	No	Hypopigmented lesions, thyroiditis
P51	*/*	/	/	1 (Female)	/	Infantile	<6	Spastic diplegia	Yes	<50	Yes	Hypochromic lesions on trunk

* Novel applies to variants never described in the literature. ** Clinical onset was defined according to Livingston [5] in: Prenatal/neonatal onset, infantile onset (onset presenting in the first few months of life) and later onset (onset beyond the first year of life). ^#^ Clinical score ≥ 12 corresponds to severe disability, a score between 6 and 12 to moderate disability and a score ≤ 6 to mild disability. ° General Quotient (GQ)/Intelligence Quotient (IQ) were measured, whenever possible, using standardized tests appropriate for age (Griffiths’ Developmental Scale, Wechsler Preschool and Primary Scale of Intelligence (WPPSI-R) or WISC-R [33,34,35]. In many cases GQ/IQ was not evaluable, due to clinical severity. ^†^ Siblings.

**Table 2:** Summary of identified mutations.

Gene	Mutation	Homozygous/Heterozygous ^%^	MAF ^£^ (ExAC)
***TREX1***	p.S88Kfs*	0/1	NA
	p.R97H	0/1	T = 0.000008/1
	p.R114H	1/1	A = 0.0002/19
	p.N51Gfs*50	1/0	NA
	p.P290_A295del	0/1	- = 0.00007/8
	p.R169H	0/1	A = 0.0002/19
***RNASEH2A***	p.R108W	0/1	A = 0.000008/1
	p.F230L	0/1	A = 0.000008/1
	p.R186W	0/1	NA
	p.V23V	0/1	A = 0.00002/3
***RNASEH2B***	p.W73L	0/2	NA
	p.T163I	0/7	NA
	p.A177T	17/12	A = 0.0013/158
	p.V185G	1/0	NA
	p.A212V	0/1	NA
	p.Ex9_Ex11del	0/1	NA
	c.64+1G>A	0/1	NA
***RNASEH2C***	p.D39Y	0/1	NA
	c.173-1G>C	0/1	
***SAMHD1***	p.D137G	2/0	T = 0.000008/1
	p.Q465*	0/1	NA
	c.1410+5G>C	0/1	NA
	p.Ex12_Ex16del	1/0	NA
***ADAR1***	p.P193A	0/1	C = 0.0021/260
	p.A870T	0/1	NA
***IFIH1***	p.D393V	0/1	C = 0.000008/1
	p.R720Q	0/1	NA
	p.R824K	0/1	NA
	p.R779H	0/2	NA
	p.M854K	0/1	NA
***RNASET2***	p.K133del	0/1	- = 0.00002/2
	p.E49*	0/1	NA

^%^ Heterozygous/Homozygous: Number of families with a heterozygous/homozygous change. ^£^ MAF: Frequency of the allele/number of times the SNP has been observed in the studied population.
